# Clonal Distribution of Invasive Pneumococci, Czech Republic, 1996–2003

**DOI:** 10.3201/eid1602.080535

**Published:** 2010-02

**Authors:** Helena Žemličková, Pavla Urbášková, Vladislav Jakubů, Jitka Motlová, Martin Musílek, Bohumír Procházka

**Affiliations:** National Institute of Public Health, Prague, Czech Republic

**Keywords:** Streptococcus pneumoniae, pneumococcal infections, molecular epidemiology, pneumococcal vaccines, bacteria, Czech Republic, dispatch

## Abstract

We conducted surveillance on invasive pneumococci isolated from adults in the Czech Republic during 1996–2003. The 7 most prevalent serotypes were characterized. Coverage with the 7-valent pneumococcal conjugate vaccine was low. Our observations confirm that detection methods may have modified the expected effect of this vaccine.

*Streptococcus pneumoniae* is a leading cause of illness and death in children and adults (*1**,**2*). The incidence of invasive pneumococcal disease (IPD) has been reduced by a 7-valent pneumococcal conjugate vaccine (PCV7) (*3*). Although certain serotypes of *S*. *pneumoniae* are recognized among children, adults carry a wider range of serotypes (*4*). Several studies have reported cases of IPD in adults caused by common childhood serotypes (*5*,*6*). The causes of such cases are unclear, but use of antimicrobial drugs (*6*), progressive population aging (*5*), and modifications to blood culture protocols have been suggested (*7*). PCV7 efficacy in children has raised the question of whether to use PCV7 in elderly persons (*5*).

In the Czech Republic, patients in follow-up care departments (i.e., <1% of the adult population), have been vaccinated with a 23-valent polysaccharide vaccine since 2001. PCV7 showed a reported 66% serotype coverage in children (*8*) and has been included in the routine childhood immunization program since January 2009.

Clones associated with IPD have been characterized by genotyping. Use of PCV7 exerts selective pressure against vaccine serotypes. Clonality data obtained before vaccine use will make it possible to demonstrate future changes in the genetic structure of pneumococci. Therefore, the purpose of our study was to identify IPD serotype distribution and clonality in adults in the Czech Republic before the use of PCV7.

## The Study

Invasive pneumococcal isolates from adults >18 years of age were obtained from blood (n = 460) and cerebrospinal fluid (CSF) (n = 229) during 1996–2003 by 54 laboratories serving ≈85% of the population in the Czech Republic. Isolates were serotyped by using the Quellung reaction (*9*). Antimicrobial drug susceptibility and MICs were determined by the broth microdilution method according to Clinical and Laboratory Standards Institute recommendations (*10*). Genotyping was performed by using pulsed-field gel electrophoresis (PFGE) (*11*) and multilocus sequence typing (MLST) (*12*). MLST results were identical between isolates of the same serotype within a PFGE subtype (*13*). Cluster analyses were based on allelic data that used the eBURST algorithm (*14*). Numerical trend was evaluated by regression analysis of the logarithm of the number of isolates by using SPSS for Windows version 14 (SPSS, Inc., Chicago, IL, USA). The χ^2^ test for trend (Epi Info version 3.4; Centers for Disease Control and Prevention, Atlanta, GA, USA) was used to evaluate variations in proportions of different serotypes; p values <0.05 were considered significant.

**Figure 1:**
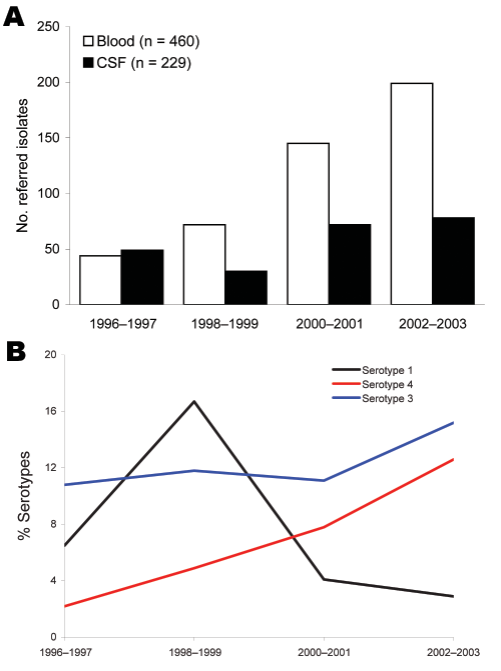
A) Invasive pneumococcal isolates from blood and cerebrospinal fluid (CSF) and B) frequency of *Streptococcus pneumoniae* serotypes 1, 3, and 4 among adults, Czech Republic, 1996–2003.

**Figure 2:**
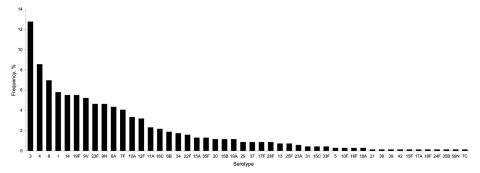
Distribution of pneumococcal invasive serotypes among 689 adults, Czech Republic, 1996–2003.

During 1996–2003, a total of 446 (64.7%) isolates were obtained from adults 18–64 years of age and 243 (35.3%) isolates were obtained from adults >65 years of age. A progressive increase in recovery of isolates from blood was observed from 44 during 1996–1997 to 199 during 2002–2003 (p = 0.009) (Figure 1). Serotyping identified 48 serotypes; 10 of these serotypes included >30 isolates. Half of all strains belonged to serotypes 3 (12.7%), 4 (8.6%), 8 (7%), 1 (5.8%), 19F (5.5%), 14 (5.5%), and 9V (5.2%) (Figure 2). Serotype coverage rates by PCV7, PCV10, PCV13, and 23-valent polysaccharide vaccine were 32.5%, 44.2%, 62.3%, and 82.1%, respectively, among adults 18–64 years of age and 35.4%, 42.8%, 61.3%, and 85.6%, respectively, among adults >65 years of age.

Serotype distribution did not vary significantly with age group and type of isolate, except for serotype 1, which was more common (p = 0.037) among younger adults (18–64 years) and more prevalent in blood (p = 0.004). With the exception of 1998–1999, serotype 3 was most common identified. This serotype, which composed≈11% of all isolates during 1996–2001, increased to 15% during 2002–2003 (p = 0.289). A change was also observed in the proportions of serotypes 1 and 4 (Figure 1). Serotype 1 increased from 6% during 1996–1997 to 17% during 1998–1999 (p = 0.027) and then decreased to 3% during 2002–2003 (p<0.001). Serotype 4 increased from 2% during 1996–1997 to 13% during 2002–2003 (p<0.001) (Figure 1).

Penicillin-resistant pneumococci were uncommon (range 1%–5%), and no isolate showed high resistance to penicillin (MIC >1 mg/L). Resistance to chloramphenicol decreased from 5% during 1996–1997 to 2% during 2002–2003 (p = 0.114). Tetracycline resistance also decreased from 8% during 1996–1997 to 6% during 2002–2003 (p = 0.732). Few (<9) isolates were resistant to erythromycin. Antimicrobial drug resistance was associated with certain serotypes. Eighteen of 23 penicillin-resistant pneumococci isolates belonged to serotype 9V. Resistance to chloramphenicol, usually observed with resistance to tetracycline, was seen most often in serotype 19F isolates.

Of 347 isolates of the 7 most common serotypes, 335 were characterized by PFGE and 130 PFGE subtypes were analyzed by MLST (Appendix Table). Forty-six allelic profiles were identified, 18 of which carried new alleles (n = 11) or novel combinations of known alleles (n = 7). Of 10 new alleles, 5 belonged to serotype 4. The *gdh128* allele was identified in serotype 4 and 9V isolates. Until recently, only *gdh128* was found among isolates from other countries. At least 60% of isolates of serotypes 1, 3, 9V, 14, and 19F have been identified within clones in the Pneumococcal Molecular Epidemiology Network. In contrast, only 25% of serotype 4 and 8 isolates were identified in this network, and these serotypes consisted of larger clusters unrelated to global clones.

## Conclusions

When we compared our results with those of a study from the United States (*5*), where 51% of IPD was among elderly persons before introduction of PCV7 and caused by pediatric serotypes (6B, 9V, 14, 19F, and 23F) (*5*), our data showed a low frequency (27.2%) of pediatric serotypes in older adults. The low frequency of penicillin-nonsusceptible pneumococci isolates of pediatric serotypes explains the observed predominance of drug-susceptible serotypes. Local differences in blood culture protocols could also bias the serotype distribution (*7*). Although the number of blood cultures performed in the Czech Republic is increasing, lower numbers of invasive pneumococci have been isolated than in other countries in Europe (www.rivm.nl/earss).

The observed predominance of serotype 3, previously reported as less invasive but associated with a high death rate (*15*), could be explained by sample collection primarily from the most severe IPD cases. In 1998, an increase in serotype 1 invasive disease was reported in Sweden. In our study, serotype 1 was the most common during the same period. Subsequently, the frequency of this serotype decreased but the frequency of bacteremia increased. The gradual increase in bacteremia and increased frequency of serotype 4, previously found to be moderately invasive (*7*), could be ascribed to the increase in blood cultures that facilitated detection of moderate disease caused by serotype 4 rather than dissemination of a specific invasive clone because the isolates showed high genotypic diversity. In our collection of invasive strains, the frequency of antimicrobial drug–resistant global clones, all previously found in the Czech Republic (*13*), varied between serotypes.

Our results support the need for IPD surveillance because differences in detection methods might modify the effect of the vaccine. Continued surveillance will be helpful in detecting any shift in the distribution of invasive serotypes and clones circulating in the Czech Republic and in monitoring the effect of PCV7 on serotype frequencies and the genetic structure of pneumococci.

## Supplementary Material

Appendix Table Genotypic data of invasive pneumococcal serotypes among adults, Czech Republic, 1996-2003*
