# Supplementary material for: Clonal Distribution of Invasive Pneumococci, Czech Republic, 1996–2003
Source: Emerg Infect Dis. 2010 Feb;16(2):287–9. doi: 10.3201/eid1602.080535 (PMC2957987; doi:10.3201/eid1602.080535)
Supplement: Appendix Table — Genotypic data of invasive pneumococcal serotypes among adults, Czech Republic, 1996-2003* [file 08-0535_appT-s1.pdf]

Publisher: CDC; Journal: Emerging Infectious Diseases
Article Type: Dispatch; Volume: 16; Issue: 2; Year: 2010; Article ID: 08-0535
DOI: 10.3201/eid1602.080535; TOC Head: Dispatch

Appendix Table. Genotypic data of invasive pneumococcal serotypes among adults, Czech Republic, 1996–2003*

Serotype	No.	PFGE type	No. isolates in PFGE type	STs in PFGE types	No. isolates selected for MLST	MLST allelic profile							Estimated % isolates in cluster (no. isolates)	Antimicrobial drug resistance profile of isolates sharing the same ST					PMEN clone name
						aroE	*gdh*	*gki*	*recP*	*spi*	*xpt*	*ddl*		Pen	Ery	Cmp	Tet	Cot	
3	85	3_1	83	180	6	7	15	2	10	6	1	22	60 (50)	S	S	S	S	S	Netherlands[3]-31
				505	3	46	8	2	10	6	1	22		S	S	S	S	S	
				232	4	13	9	15	14	10	16	1	36 (31)	S	S	S	S	S	
				378	3	13	9	15	14	10	16	19		S	S	S	S	S	
				**2022**	1	13	9	15	14	10	**186**	1		S	S	S	S	S	
				260	2	26	9	15	14	9	16	19	2 (2)	S	S	S	S	S	
		3_2	1	458	1	2	32	9	47	6	21	17	1 (1)	S	S	S	S	S	
		3_3	1	1116	1	1	26	28	11	13	1	14	1 (1)	S	S	S	S	S	
4	58	4_1	17	800	2	8	41	4	1	6	116	6	29 (17)	S	S	S	S	S	
				801	4	8	70	4	1	6	116	6		S	S	S	S	S	
				**2361**	2	8	8	4	1	6	116	6		S	S	S	S	S	
				**2617**	2	8	70	4	16	6	116	6		S	S	S	S	S	
		4_2	14	205	9	10	5	4	5	13	10	18	24 (14)	S	S	S	S	S	Sweden[4]-38
				**2333**	1	10	5	4	5	13	**194**	18		S	S	S	S	S	
				**2388**	1	10	5	4	5	**138**	10	18		S	S	S	S	S	
				1022	1	8	8	4	1	15	1	20		S	S	S	R	S	
		4_3	7	247	1	16	13	4	5	6	10	14	12 (7)	S	S	S	S	S	
				**2342**	4	16	**141**	4	5	6	10	14		S	S	S	S	S	
				**2362**	1	16	**141**	4	4	6	10	18		S	S	S	S	S	
				**2389**	1	16	**143**	4	5	6	10	14		S	S	S	S	S	
		4_4	9	1022	3	8	8	4	1	15	1	20	16 (9)	S	S	S	R(7)/S	S	
				**2333**	1	10	5	4	5	13	**194**	18		S	S	S	S	S	
		4_5	1	**2546**	1	16	13	6	5	10	**214**	**242**	3 (2)	S	S	S	S	S	
		4_6	1	**2546**	1	16	13	6	5	10	**214**	**242**		S	S	S	S	R	
		4_7	6	**2341**	3	7	**128**	4	1	15	116	72	14 (8)	S	S	S	S	S	
		4_8	2	**2341**	1	7	**128**	4	1	15	116	72		S	S	S	S	S	
		4_9	1	1637	1	10	13	53	5	13	10	18	2 (1)	S	S	S	S	S	
8	46	8_1	17	404	2	7	9	15	11	42	1	70	78 (36)	S	S	S	S	S	
		8_2	19	404	3	7	9	15	11	42	1	70		S	S	S	S	S	
				1480	4	7	9	15	11	93	1	70		S	S	S	S	S	
				**2544**	1	7	9	15	11	93	1	14		S	S	S	S	S	
		8_3	10	53	7	2	5	1	11	16	3	14	22 (10)	S	S	S	S	S	Netherlands[8]-33
1	37	1_1	5	304	2	13	8	13	5	14	4	8	14 (5)	S	S	S	S	R(1)/S	Sweden[1]-40
				305	2	13	8	13	5	17	4	28		S	S	S	R	S	
		1_2	31	306	3	12	8	13	5	16	4	20	83 (31)	S	S	S	S	S	Sweden[1]-28

Publisher: CDC; Journal: Emerging Infectious Diseases
Article Type: Dispatch; Volume: 16; Issue: 2; Year: 2010; Article ID: 08-0535
DOI: 10.3201/eid1602.080535; TOC Head: Dispatch

		1_3	1	**2028**	1	7	31	4	34	6	4	94	3 (1)	S	S	S	S	S	
19F	36	19F_1	30	423	3	1	5	4	12	5	3	8	83 (30)	S	S	R(23)/S	R(22)/S	R(2)/S	
				**2024**	1	1	5	**141**	12	5	3	8		S	S	S	S	S	
				1960	1	1	5	4	6	5	3	8		S	S	R	R	S	
		19F_2	1	271	1	4	16	19	15	6	20	26	3 (1)	I	R	S	R	R	
		19F_3	1	**2030**	1	8	10	9	8	17	1	17	3 (1)	S	S	S	S	S	
		19F_4	2	251	1	18	2	22	1	9	23	14	8 (3)	S	S	S	S	R(1)/S	
		19F_5	1	251	1	18	2	22	1	9	23	14		S	S	S	S	S	
		19F_6	1	1551	1	5	7	4	2	10	1	6	3 (1)	S	S	S	S	S	
14	38	14_1	37	124	14	7	5	1	8	14	11	14	97 (37)	S	S	S	S	S	Netherlands^{14}-35
		14_5	1	**1772**	1	8	37	4	5	1	3	8	3 (1)	S	R	S	R	S	
9V	35	9V_1	26	156	10	7	11	10	1	6	8	1	77 (27)	I	R(1)/S	S	S	R	Spain^{9V}-3
				162	3	7	11	10	1	6	8	14		S	S	S	S	R(3)/S	
				**2025**	1	**88**	11	10	1	6	8	14		I	S	S	S	R	
				**2026**	1	7	**128**	10	1	6	8	14		S	S	S	S	R	
		9V_4	1	162	1	7	11	10	1	6	8	14		S	R	S	S	R	
		9V_2	8	239	4	15	17	4	16	6	19	17	23 (8)	S	S	S	S	R(6)/S	
				**1779**	1	15	17	4	16	6	19	14		S	S	S	S	R	
Total	335				132														

*PFGE, pulsed-field gel electrophoresis; ST, sequence type; MLST, multilocus sequence typing; *aroE*, shikimate dehydrogenase; *gdh*, glucose-6-phosphate dehydrogenase; *gki*, glucose kinase; *recP*, transketose; *spi*, signal peptidase I; *xpt*, xanthine phosphoribosyltransferase; *ddl*, D-alanine-D-alanine ligase; Pen, penicillin; Ery, erythromycin; Cmp, chloramphenicol; Tet, tetracycline; Cot, cotrimoxazole; PMEN, Pneumococcal Molecular Epidemiology Network; S, susceptible; R, resistant. New sequence types and new alleles are shown in **boldface**.
†Defined by e-BURST.
